# A biregional survey and review of first-line treatment failure and second-line paediatric antiretroviral access and use in Asia and southern Africa

**DOI:** 10.1186/1758-2652-14-7

**Published:** 2011-02-09

**Authors:** TREAT Asia Pediatric HIV Observational Database (TApHOD), The International Epidemiologic Databases to Evaluate AIDS (IeDEA) Southern Africa Paediatric Group

**Affiliations:** 1 TREAT Asia/amfAR - The Foundation for AIDS Research, Bangkok, Thailand; 2 School of Public Health and Family Medicine, University of Cape Town, Cape Town, South Africa; 3 Institute of Social and Preventive Medicine, University of Bern, Bern, Switzerland

## Abstract

**Background:**

To better understand the need for paediatric second-line antiretroviral therapy (ART), an ART management survey and a cross-sectional analysis of second-line ART use were conducted in the TREAT Asia Paediatric HIV Observational Database and the IeDEA Southern Africa (International Epidemiologic Databases to Evaluate AIDS) regional cohorts.

**Methods:**

Surveys were conducted in April 2009. Analysis data from the Asia cohort were collected in March 2009 from 12 centres in Cambodia, India, Indonesia, Malaysia, and Thailand. Data from the IeDEA Southern Africa cohort were finalized in February 2008 from 10 centres in Malawi, Mozambique, South Africa and Zimbabwe.

**Results:**

Survey responses reflected inter-regional variations in drug access and national guidelines. A total of 1301 children in the TREAT Asia and 4561 children in the IeDEA Southern Africa cohorts met inclusion criteria for the cross-sectional analysis.

Ten percent of Asian and 3.3% of African children were on second-line ART at the time of data transfer. Median age (interquartile range) in months at second-line initiation was 120 (78-145) months in the Asian cohort and 66 (29-112) months in the southern African cohort. Regimens varied, and the then current World Health Organization-recommended nucleoside reverse transcriptase combination of abacavir and didanosine was used in less than 5% of children in each region.

**Conclusions:**

In order to provide life-long ART for children, better use of current first-line regimens and broader access to heat-stable, paediatric second-line and salvage formulations are needed. There will be limited benefit to earlier diagnosis of treatment failure unless providers and patients have access to appropriate drugs for children to switch to.

## Background

An estimated 2.5 million children worldwide are living with HIV [1], and more than 354,000 of them were on antiretroviral treatment (ART) at the end of 2009. Global paediatric treatment coverage is estimated at 28% after applying revised World Health Organization (WHO) ART initiation criteria [1,2]. Once children are on treatment, the cumulative risk of treatment failure continues to increase over time. Social support, nutritional supplements, counselling, medication records, home-based care and transportation reimbursements are only a few of the many resources that are used to promote adherence to delay this outcome.

Despite being in early stages of the paediatric ART scale up, children are already failing treatment and needing second-line regimens. Current United Nations estimates of paediatric second-line use in low- and middle-income countries (LMICs; outside of the Americas) are around 3% [1]. However, this is much lower than in single cohort reports in settings where ART has been available for longer periods of time. A survey of 17 centres in LMICs in Asia reported that 20% of the more than 3600 children under care were already past their first ART regimens [3]. Other single-institution cohorts have reported as much as 5.8% (South Africa [4]), 9% (Thailand [5]), and 19.4% (south India [6]) of patients switching to second-line regimens.

Evidence-based strategies for selecting second-line regimens are needed, but are also dependent on local antiretroviral (ARV) options. Children have consistently faced greater disadvantages with regards to the availability of ARV formulations that can be dosed and delivered to children, and that are safe to use during growth and development [7,8].

To better understand the growing need for paediatric second-line ART, we conducted a survey of two regional cohorts - in Asia and southern Africa - to determine second-line use and ARV access, and compare nationally recommended regimens to explore how regimen sequencing is being approached in these regions.

## Methods

### Survey

A survey regarding second-line ART use was conducted in the TREAT Asia Pediatric HIV Observational Database (TApHOD) and the International Epidemiologic Databases to Evaluate AIDS (IeDEA) Southern Africa regional cohorts in April 2009. TApHOD was established in 2008 and includes 16 clinical centres in six countries, 12 of which currently submit patient-level data to the cohort study (Table 1). The programme is coordinated by TREAT Asia/amfAR (Bangkok, Thailand) with data management support from the National Centre in HIV Epidemiology and Clinical Research (NCHECR; Sydney, Australia). IeDEA Southern Africa was formed in 2007, and includes 10 clinical sites that provide ART for children in four countries, all of which submit data to their cohort study. It is a research collaboration coordinated by the University of Cape Town (South Africa) and the University of Bern (Switzerland).

**Table 1:** First- and second-line antiretroviral therapy regimens in use in TREAT Asia and IeDEA Southern Africa

Site	Country	Nationally recommended paediatric first-line ART regimen*	Nationally recommended paediatric second-line ART regimen after NNRTI	Most commonly used second-line regimen in current site cohort
**TREAT Asia**				

National Center for HIV, AIDS, Dermatology, and Sexually Transmitted Infections	Cambodia	d4T or AZT+3TC+NVP or EFV If NNRTI exposure: d4T+3TC+LPV/r	ABC+ ddI+LPV/r	ABC+3TC+LPV/r

Beijing Ditan Hospital	China	d4T or AZT+3TC+NVP or EFV If NNRTI exposure: AZT+3TC+LPV/r	ABC+3TC+AZT+LPV/r	ABC+3TC+AZT+LPV/r

YRG Centre for AIDS Research and Education	India	d4T or AZT+3TC+NVP or EFV	<3 yr: ABC+ddI+3TC+LPV/r >3 yr: TDF+3TC or FTC+LPV/r	TDF or ddI+3TC or FTC+LPV/r

Cipto Mangunkusumo Hospital	Indonesia	d4T or AZT+3TC+NVP or EFV	ddI+3TC+LPV/r	ddI+3TC+LPV/r

Hospital Kuala Lumpur	Malaysia	<3 yr: AZT+3TC or ddI+NVP ≥3 yr: AZT+3TC or ddI+EFV	2 new NRTI+LPV/r	d4T+3TC+LPV/r

Hospital Likas	Malaysia	<3 yr: AZT+3TC or ddI+NVP ≥3 yr: AZT+3TC or ddI+EFV	2 new NRTI+LPV/r	d4T+ddI+LPV/r

Hospital Penang	Malaysia	<3 yr: AZT+3TC or ddI+NVP ≥3 yr: AZT+3TC or ddI+EFV	2 new NRTI+LPV/r	--

Hospital Raja Perempuan Zainab	Malaysia	<3 yr: AZT+3TC or ddI+NVP ≥3 yr: AZT+3TC or ddI+EFV	2 new NRTI+LPV/r	d4T+ddI+LPV/r

Chiang Mai University Medical Centre	Thailand	d4T or AZT+3TC+NVP or EFV	ddI+ABC or 3TC+PI/r	AZT+3TC+LPV/r

Chiang Rai Regional Hospital	Thailand	d4T or AZT+3TC+NVP or EFV	ddI+ABC or 3TC+PI/r	AZT+3TC+LPV/r

HIV-NAT	Thailand	d4T or AZT+3TC+NVP or EFV	ddI+ABC or 3TC+PI/r	AZT+3TC+LPV/r

Khon Kaen University Medical Centre	Thailand	d4T or AZT+3TC+NVP or EFV	ddI+ABC or 3TC+PI/r	AZT+ddI+LPV/r

Siriraj Hospital	Thailand	d4T or AZT+3TC+NVP or EFV	ddI+ABC or 3TC+PI/r	AZT+ddI+LPV/r

Children's Hospital 1	Vietnam	d4T+3TC+NVP	ABC+ddI+LPV/r	--

Children's Hospital 2	Vietnam	d4T+3TC+NVP	ABC+ddI+LPV/r	ABC+ddI+LPV/r

National Hospital of Pediatrics	Vietnam	d4T+3TC+NVP	ABC+ddI+LPV/r	ABC+ddI+LPV/r

**IeDEA Southern Africa**				

Lighthouse Clinic	Malawi	d4T+3TC+NVP	ABC+ddI+LPV/r	AZT+3TC+LPV/r

Paediatric Day Hospital, Maputo	Mozambique	d4T or AZT+3TC+NVP or EFV If NNRTI exposure: d4T or AZT+3TC+LPV/r	ABC+ddI+LPV/r	None

Rahima Moosa Mother and Child Hospital	South Africa	<3 yr/10 kg: d4T+3TC+LPV/r >3 yr/10 kg: d4T+3TC+EFV	AZT+ddI+LPV/r	ABC+3TC+LPV/r

Gugulethu Community Health Centre	South Africa	<3 yr/10 kg: d4T+3TC+LPV/r >3yr/10kg: d4T+3TC+EFV	AZT+ddI+LPV/r	AZT+ddI+EFV or LPV/r ABC+3TC+LPV/r

Harriet Shezi Clinic	South Africa	<3 yr/10 kg: d4T+3TC+LPV/r >3 yr/10 kg: d4T+3TC+EFV	AZT+ddI+LPV/r	AZT+ddI+LPV/r

Khayelitsha Community Health Centre	South Africa	< 3 yr/10 kg: d4T+3TC+LPV/r >3 yr/10 kg: d4T+3TC+EFV	AZT+ddI+LPV/r	AZT+ddI+LPV/r

McCord Hospital	South Africa	<3 yr/10 kg: d4T+3TC+LPV/r >3 yr/10 kg: d4T+3TC+EFV	AZT+ddI+LPV/r	AZT+ddI+LPV/r

Red Cross Children's Hospital	South Africa	<3 yr/10 kg: d4T+3TC+LPV/r >3 yr/10 kg: d4T+3TC+EFV	AZT+ddI+LPV/r	AZT+ddI+EFV

Tygerberg Hospital	South Africa	<3 yr/10 kg: d4T+3TC+LPV/r >3 yr/10 kg: d4T+3TC+EFV	AZT+ddI+LPV/r	AZT+ddI+LPV/r

Newlands Clinic	Zimbabwe	AZT+3TC+NVP	ddI+3TC+LPV/r	d4T+3TC+NVP

*Content reflects current recommendations at the time of the survey. WHO first-line regimen recommendations at the time of the survey included two NRTIs with one NNRTI or two NRTIs with one PI/r if the infant had previous NNRTI exposure [10,17]; second-line regimen recommendations after NNRTI failure included two NRTIs with one PI/r or unboosted nelfinavir in limited circumstances.

d4T - stavudine; AZT - zidovudine; 3TC - lamivudine; NVP - nevirapine; EFV - efavirenz; ABC - abacavir; ddI - didanosine; LPV/r - ritonavir-boosted lopinavir; TDF - tenofovir; FTC - emtricitabine; NRTI - nucleoside reverse transcriptase inhibitor; NNRTI - non-nucleoside reverse transcriptase inhibitor.

Site-level questions queried access to nucleoside/nucleotide reverse transcriptase inhibitors (NRTI/NtRTIs) and protease inhibitors (PIs) commonly used in internationally recommended ART regimens. Drug access was designated as "easy" (i.e., regular and consistent access and supply), "somewhat difficult" (i.e., occasional difficulties in accessing and/or obtaining), "difficult" (i.e., frequent difficulties in accessing and/or obtaining), or "cannot or do not access" (i.e., drug was not available or clinicians did not use). Nationally recommended paediatric ART regimens were obtained from individual country guidelines, when available, or by self-report from site principal investigators.

### Observational cohort data

In each regional cohort study, participating sites submit anonymized, patient-level data to their regional data management centres for data cleaning and analysis. TApHOD data are submitted twice a year. Data included in this survey were collected in March 2009 from 12 centres in Cambodia, India, Indonesia, Malaysia and Thailand. Data from the IeDEA Southern Africa cohort were finalized in February 2008 from 10 centres in Malawi, Mozambique, South Africa and Zimbabwe.

Cross-sectional data on first- and second-line ART use in children who were alive, on ART, and actively under care as of the data submission date were eligible for inclusion. Children in the cohort who had previously been documented to have died, been transferred out of the site, or were lost to follow up were consequently excluded from the analysis.

In order to more accurately reflect clinical outcomes with current paediatric ART management practices using highly active three-drug regimens and to avoid potential misclassification of second-line regimens, children also were excluded in the following circumstances: 1) first ART regimens were unknown or missing from the database; 2) first ART regimens were either mono- or dual-therapy; or 3) first ART regimens contained didanosine. Children using didanosine were excluded in order for the analysis cohort to more closely reflect standard, WHO-recommended first-line regimens [9].

Second-line switches were defined as a change in two or more ARVs, including a class switch, i.e., from non-nucleoside reverse transcriptase inhibitors (NNRTIs) to PIs or visa versa, or if a single-drug class switch was made on the basis of reported treatment failure; regimens could not be reverted for at least 24 weeks to avoid including changes due to temporary stock outs. Descriptive statistics were conducted in SAS and STATA.

## Results

All sites in TREAT Asia (n = 16) and IeDEA Southern Africa (n = 10) responded to the survey (Table 1). Nationally recommended first-line ART regimens were consistent with WHO guidelines, and were most commonly combinations of stavudine or zidovudine with lamivudine and nevirapine or efavirenz. The four Malaysian hospitals allowed for the use of didanosine in the first-line NRTI backbone. In addition, boosted PIs were recommended in some centres when previous NNRTI exposure was known (Cambodia, China, Mozambique). South Africa recommended a boosted PI for first-line treatment of all children under three years of age or weighing less than 10 kilograms.

All recommended second-line regimens for children failing NNRTI-based first-line ART included ritonavir-boosted lopinavir. The NRTI combination varied from the WHO-recommended combination of abacavir and didanosine to substituting with another thymidine analogue (i.e., zidovudine for stavudine). A third of the sites continued lamivudine in second-line regimens. Malaysian and South African sites, representing 42% of all sites, had specific national recommendations for second-line regimens after initial PI failure that advised use of two new NRTIs and either nevirapine or efavirenz (data not shown).

There was some variability in levels of drug access between the regions (Table 2). IeDEA Southern Africa had better access to abacavir and ritonavir suspension; TREAT Asia sites had better access to tenofovir and paediatric, heat-stable boosted lopinavir tablets. Although all of the African sites had access to the adult version of the boosted lopinavir, 80% found it either difficult to access the liquid or paediatric formulation of the tablet, or did not use it for their patients. This was related to delays at the time of the survey in the drug's approval by the South African Medicines Control Council that have since been resolved.

**Table 2:** Levels of access to commonly used second-line antiretroviral drugs in TREAT Asia and IeDEA Southern Africa

	Categories of drug access							
Antiretroviral*	**Easy to access**		**Somewhat difficult to access**		**Difficult to access**		**Cannot or do not access**	

	**TA**	**SA**	**TA**	**SA**	**TA**	**SA**	**TA**	**SA**

ABC	38%	60%	6%	0	19%	30%	38%	10%

ddI	100%	90%	0	0	0	0	0	10%

TDF	50%	10%	38%	30%	0	10%	13%	50%

ATV	19%	10%	13%	0	6%	0	63%	90%

IDV	56%	10%	6%	10%	6%	20%	31%	60%

LPV/r, liquid	69%	80%	13%	20%	0	0	19%	0

LPV/r, paediatric tablet	50%	10%	0	10%	19%	10%	31%	70%

LPV/r, adult tablet	81%	100%	0	0	13%	0	6%	0

LPV/r, adult capsule	31%	60%	19%	20%	6%	0	44%	20%

NFV	6%	20%	13%	0	0	20%	81%	60%

RTV, liquid	13%	50%	19%	20%	19%	0	50%	30%

RTV, capsule	38%	50%	13%	30%	0	0	50%	20%

SQV	13%	10%	13%	10%	25%	30%	50%	50%

*any formulation unless noted otherwise

TA - TREAT Asia; SA - IeDEA Southern Africa; ABC - abacavir; ddI - didanosine; TDF - tenofovir; ATV - atazanavir; IDV - indinavir; LPV/r - ritonavir-boosted lopinavir; NFV - nelfinavir; RTV - ritonavir; SQV - saquinavir.

A total of 1301 children in the TREAT Asia and 4561 children in the IeDEA Southern African cohorts met inclusion criteria for the cross-sectional analysis (Tables 3 and 4). Although stavudine was infrequently used in Asia, it was part of the first-line regimen in 92% of children in southern Africa. Ten percent of Asian and 3.3% of African children were on second-line ART at the time of data transfer. Asian children were on second-line ART for longer periods and were older at the time of switch. African children on second-line ART were more frequently male (67%). Regimens varied widely, and the WHO-recommended combination at the time of the data transfer of abacavir and didanosine [10] was used in less than 5% of children in each region.

**Table 3:** Paediatric antiretroviral therapy (ART) utilization among children on first-line ART at data transfer in TREAT Asia and IeDEA Southern Africa

TREAT Asia (N = 1164)			IeDEA Southern Africa (N = 4412)	
Female, N (%)	608 (52)		Female, N (%)	2179 (49)

Most common regimens, N (%)			Most common regimens	
AZT+3TC+NVP	529 (46)		d4T+3TC+EFV	2154 (49)
AZT+3TC+EFV	299 (26)		d4T+3TC+LPV/r	979 (22)
d4T+3TC+NVP	183 (16)		d4T+3TC+NVP	671 (15)
d4T+3TC+EFV	53 (5)		d4T+3TC+LPV/r+RTV	128 (3)
AZT+3TC+LPV/r	33 (3)		AZT+3TC+NVP	119 (3)

Median age, months (IQR) at start	85 (47-119)		Median age, months (IQR) at start	56 (22-96)

Median age, months (IQR) at data transfer	129 (90-163)		Median age, months (IQR) at data transfer	79 (43-119)

Median months (IQR) on regimen	38 (20-58)		Median months (IQR) on regimen	19 (9-31)

**Table 4:** Paediatric antiretroviral therapy (ART) utilization among children on second-line ART at data transfer in TREAT Asia and IeDEA Southern Africa

TREAT Asia (N = 137)			IeDEA Southern Africa (N = 149)	
Female, N (%)	69 (50)		Female, N (%)	49 (33)

Most common regimens, N (%)			Most common regimens	
AZT+3TC+LPV/r	31 (23)		AZT+ddI+LPV/r	54 (36)
AZT+ddI+LPV/r	29 (21)		AZT+ddI+EFV	17 (11)
ddI+3TC+LPV/r	15 (11)		AZT+TDF+LPV/r	14 (9)
LPV/r+IDV	8 (6)		ABC+3TC+LPV/r	12 (8)
d4T+ddI+LPV/r	7 (5)		d4T+3TC+LPV/r	8 (5)

Median age, months (IQR) at start	120 (78-145)		Median age, months (IQR) at start	66 (29-112)

Median age, months (IQR) at data transfer	146 (102-173)		Median age, months (IQR) at data transfer	104 (62-143)

Median months (IQR) on regimen	17 (9-38)		Median months (IQR) on regimen	12 (4-18)

AZT - zidovudine; 3TC - lamivudine; NVP - nevirapine; d4T - stavudine; EFV - efavirenz; LPV/r - ritonavir-boosted lopinavir; ddI - didanosine; IDV - indinavir; ABC - abacavir; TDF - tenofovir

## Discussion

The percentage of children on second-line in the IeDEA Southern Africa cohort was similar to United Nations estimates, but it was three times higher in the TREAT Asia cohort. Although southern African data were collected one year earlier, this marked difference may be related to the longer history of paediatric ART in Asia relative to the more recent scale up observed in southern Africa.

In fact, we are likely to have underestimated the use of second-line ART in the Asian cohort, since 18% of children excluded from this analysis had a previous exposure to mono- and dual-NRTI regimens. In addition, these differences may be related to regional variation in the availability of clear second-line switch criteria, and broader access to viral load testing in Asia.

However, these data reflect only those currently on second-line treatment. Estimates for how many children are ready to switch to second-line ART now and projections for the future are critically needed in order to prepare providers, governments and donors. If the need for second-line ART is based on virologic failure alone, the numbers in need would be much higher.

In Asia, 15% of children in a Cambodian study and 37% of children in a Chinese study had viral loads of more than 1000 copies/ml after 12 months of first-line ART [11,12]. An earlier Thai cohort reported that 17% of children had virologic failure after 192 weeks of ART [5]. Similarly, a study done in southern Africa using the cut-off of more than 1000 copies/ml reported a cumulative probability of failure by three years after ART initiation of 19.3% [13], and a study of children and adolescents in Uganda reported 26% with viral loads of more than 400 copies/ml after 12 months of treatment [14].

Even in settings where viral load is routinely available, paediatricians are less inclined to switch children who have persistent viremia unless adherence to a new regimen can be assured and the benefits of a new regimen outweigh the risks of running out of ART options. Furthermore, when the initial regimen includes a boosted PI, low-level viremia may not indicate resistance to the PI. Most importantly, the decision to switch ART at a young age in countries that only have two lines of national ART regimens can leave children with no suppressive regimens by adolescence. That the median age at switch in IeDEA Southern Africa was 5.5 years was especially concerning because of the lack of available third-line options that these young children now have. This may reflect the impact of NNRTI resistance after prevention of mother to child transmission interventions, which can also be a factor in ART management after first-line PI failure in those infants who are started on boosted lopinavir.

Another notable finding was that only 33% of the children on second-line treatment in the southern African cohort were female. However in an analysis using the same data of factors that predict switch to second-line in southern Africa, gender was not predictive after adjustment for age, duration on ART, disease severity at the time of failure and first-line regimen [13].

A wide range of second-line regimens was in use. Unlike the United Nations data reporting that at least 46.7% of paediatric second-line regimens in the 59 LMICs it surveyed contained abacavir [1], this ARV was infrequently used in either the TREAT Asia or the IeDEA Southern Africa cohorts. Most of the second-line regimens included recycling of a thymidine analogue (i.e., zidovudine). It was unexpected that abacavir was more difficult to access by clinical sites in Asia despite being part of the WHO-recommended second-line regimen. The relatively higher cost of abacavir compared with zidovudine may also be a deterrent to its use. Access to a broader range of paediatric ARVs is needed in order to maximize the potency of second- and third-line regimens, whenever possible.

Another outcome of this survey was to document the differences in use of stavudine between the regions. Recent WHO recommendations for adult ART have proposed setting up plans for phasing out stavudine by 2011 because of long-term toxicity with this drug [15]. Similar recommendations for children may also be justified [16]. Scaling up of paediatric treatment in many developing countries depends on simple fixed-dose combinations and child-friendly adapted formulations, such as dispersible tablets, improved palatability and heat-stable formulations (for storage in tropical climates). Examples include the need for ritonavir-boosted atazanavir and heat-stable ritonavir-boosted lopinavir in palatable paediatric formulations.

In addition, given the difficulties of accessing clean water in many resource-limited rural settings, formulations that require reconstitution should ideally be avoided. Efforts are also needed to ensure that newer drugs, such as raltegravir, darunavir and etravirine, are also developed as heat-stable formulations and tested for use in infants and young children as these represent important potential options for both, and for improving first-line regimens and as salvage therapy.

Finally, improving access to effective paediatric treatment also requires improved access to diagnostic tools, including PCR for early infant diagnosis and more widespread access to viral load technologies for early diagnosis of treatment failure. Although rarely available, the role of resistance testing in LMIC settings continues to be unclear. Further research on when and how HIV genotyping in heavily experienced children can be cost-effective is needed to identify possible strategies for its use.

Our data are limited by their cross-sectional nature and depth. The potential impact of changes in drug access and national or global paediatric treatment guidelines are difficult to assess from our surveys and the regional databases. Additional detail on the durability of first-line regimens in the children with treatment failure is available for the southern Africa cohort in a previous publication [13], but has not yet been analyzed for the Asian cohort. The survey on drug access did not separate out drugs that could not be accessed from those there were simply not used in the clinic.

The participating clinical centres are also largely urban referral centres, preventing generalization of these results. However, these cohorts include some of the most experienced patients in these regions, who are facing challenges today that are expected to arise for all children as they age into adulthood. The lessons we are learning from these children's experiences with ART can be used to better prepare national-level programmes for the future.

## Conclusions

Although better use of first-line drugs can delay failure and improve second-line outcomes, the need for second-line paediatric ART in LMICs will continue to grow. The availability of potent, less toxic ARVs for both first- and second-line regimens must keep pace with children as they transition to adolescence and adulthood. Ultimately, there will be limited benefit to earlier diagnosis of treatment failure unless providers and patients have access to appropriate drugs for children to switch to.

## Competing interests

The authors declare that they have no competing interests.

## Authors' contributions

All authors listed below have equally contributed to the study. All authors have read and approved the final version of the manuscript.

## Authors' information

The TREAT Asia Paediatric HIV Network

V Saphonn* and S Saramony, National Centre for HIV/AIDS Dermatology and STDs, Phnom Penh, Cambodia;

U Vibol* and S Sophan, National Pediatric Hospital, Phnom Penh, Cambodia;

J Tucker, New Hope for Cambodian Children, Phnom Penh, Cambodia;

FJ Zhang and N Han, Beijing Ditan Hospital, Beijing, China;

N Kumarasamy* and S Saghayam, YR Gaitonde Centre for AIDS Research and Education, Chennai, India;

N Kurniati*‡ and D Muktiarti, Cipto Mangunkusumo General Hospital, Jakarta, Indonesia;

SM Fong* and M Thien, Hospital Likas, Kota Kinabalu, Malaysia;

NK Nik Yusoff* and LC Hai, Hospital Raja Perempuan Zainab II, Kelantan, Malaysia;

K Razali* and NF Abdul Rahman, Pediatric Institute, Hospital Kuala Lumpur, Kuala Lumpur, Malaysia;

R Nallusamy* and KC Chan, Penang Hospital, Penang, Malaysia;

V Sirisanthana*and L Aurpibul, Chiang Mai University, Chiang Mai, Thailand;

R Hansudewechakul* and A Khongponoi, Chiangrai Prachanukroh Hospital, Chiang Rai, Thailand;

P Lumbiganon* and P Kosalaraksa., Khon Kaen University, Khon Kaen, Thailand;

G Jourdain, Program for HIV Prevention and Treatment, Chiang Mai, Thailand;

J Ananworanich*† and T Suwanlerk, The Netherlands, Australia, Thailand Research Collaboration (HIV-NAT), Bangkok, Thailand;

K Chokephaibulkit* and O Wittawatmongkol, Siriraj Hospital, Mahidol University;

HK Truong* and DAN Mai, Children's Hospital 1, Ho Chi Minh City, Vietnam;

CV Do* and MT Ha, Children's Hospital 2, Ho Chi Minh City, Vietnam;

HV Bui *and VL Nguyen, National Hospital of Pediatrics, Hanoi, Vietnam;

ON Le, Worldwide Orphans Foundation, Ho Chi Minh City, Vietnam;

AH Sohn*, L Messerschmidt and J Pang, TREAT Asia, amfAR - The Foundation for AIDS Research, Bangkok, Thailand;

DA Cooper, MG Law* and A Kariminia, National Centre in HIV Epidemiology and Clinical Research, The University of New South Wales, Sydney, Australia;

*TApHOD Steering Committee member

† Current Steering Committee Chair; ‡ co-Chair

IeDEA Southern Africa Paediatric Group

Mary-Ann Davies, School of Public Health and Family Medicine, University of Cape Town, South Africa;

Brian Eley, Red Cross Children's Hospital and the School of Child and Adolescent Health, University of Cape Town, Cape Town, South Africa;

Janet Giddy, McCord Hospital, Durban, South Africa;

Harry Moultrie, Institute for Sexual Reproductive Health, HIV and Related Diseases, Faculty of Health Sciences, University of the Witwatersrand, Johannesburg, South Africa;

Margaret Pascoe, Newlands Clinic, Harare, Zimbabwe;

Helena Rabie, Tygerberg Academic Hospital and University of Stellenbosch, Cape Town, South Africa;

Karl Technau, Empilweni Service and Research Unit, Rahima Moosa Mother and Child Hospital, University of the Witwatersrand, Johannesburg, South Africa;

Gilles Van Cutsem, Khayelitsha ART Programme, Médecins Sans Frontières and School of Public Health and Family Medicine, University of Cape Town, Cape Town, South Africa;

Paula Vaz, Paediatric Day Hospital, Maputo, Mozambique;

Ralf Weigel, Lighthouse Trust, Lilongwe, Malawi;

Robin Wood, Gugulethu Community Health Centre and Desmond Tutu HIV Centre, Institute of Infectious Disease and Molecular Medicine, University of Cape Town, Cape Town, South Africa

IeDEA Southern Africa Steering Group

**Member sites: **Anna Coutsoudis, PMTCT Plus, Durban, South Africa; Diana Dickinson, Gaborone Independent Hospital, Gaborone, Botswana; Brian Eley, Red Cross Children's Hospital, Cape Town, South Africa; Lara Fairall, Free State provincial ARV roll out, South Africa; Tendani Gaolathe, Princess Marina Hospital, Gaborone, Botswana; Janet Giddy, McCord Hospital, Durban, South Africa; Timothy Meade, CorpMed Clinic, Lusaka, Zambia; Patrick MacPhail, Themba Lethu Clinic, Helen JosephHospital, Johannesburg, South Africa; Lerato Mohapi, Perinatal HIV Research Unit, Johannesburg, South Africa; Margaret Pascoe, Newlands Clinic, Harare, Zimbabwe; Hans Prozesky, Tygerberg Academic Hospital, Stellenbosch, South Africa; Harry Moultrie, Enhancing Children's HIV Outcomes (Harriet Shezi Children's Clinic, Chris Hani Baragwanath Hospital, Soweto); Karl Technau, University of Witwatersrand Paediatric HIV Clinics (Empilweni Service and Research Unit, Rahima Moosa Mother and Child Hospital), Johannesburg, South Africa; Gilles van Cutsem, Khayelitsha ART Programme and Médecins sans Frontières, Cape Town, South Africa; Paula Vaz, Paediatric Day Hospital, Maputo, Mozambique; Ralf Weigel, Lighthouse Trust, Lilongwe, Malawi; Robin Wood, Gugulethu and Masiphumelele ART Programmes, Cape Town, South Africa.

**Central team: **Matthias Egger, Beatrice Fatzer, Claire Graber and Olivia Keiser, Institute of Social and Preventive Medicine, University of Bern, Bern, Switzerland; Andrew Boulle, Morna Cornell, Mary-Ann Davies, Nicola Maxwell, Landon Myer and Anna Grimsrud, School of Public Health and Family Medicine, University of Cape Town, Cape Town, South Africa.

## References

[B1] UNAIDS UNAIDS Report on the global AIDS epidemic. Geneva 2010

[B2] WHO Towards Universal Access. Scaling up priority HIV/AIDS interventions in the health sector. Geneva 2010

[B3] Prasitsuebsai W Bowen AC Pang J Hesp C Kariminia A Sohn AH Pediatric HIV clinical care resources and management practices in Asia: a regional survey of the TREAT Asia pediatric network AIDS Patient Care STDS 2009 14 127 131 10.1089/apc.2009.0224 PMC2835391 20059355

[B4] Jaspan HB Berrisford AE Boulle AM Two-year outcomes of children on non-nucleoside reverse transcriptase inhibitor and protease inhibitor regimens in a South African pediatric antiretroviral program Pediatr Infect Dis J 2008 14 993 998 10.1097/INF.0b013e31817acf7b 18818556

[B5] Puthanakit T Aurpibul L Oberdorfer P Akarathum N Kanjanavanit S Wannarit P Sirisanthana T Sirisanthana V Sustained immunologic and virologic efficacy after four years of highly active antiretroviral therapy in human immunodeficiency virus infected children in Thailand Pediatr Infect Dis J 2007 14 953 956 10.1097/INF.0b013e318125720a 17901804

[B6] Kumarasamy N Venkatesh KK Devaleenol B Poongulali S Mothi SN Solomon S Safety, Tolerability and Effectiveness of Generic HAART in HIV-Infected Children in South India J Trop Pediatr 2009 14 155 9 10.1093/tropej/fmn080 18829638

[B7] Sohn AH Ananworanich J How can we simplify antiretroviral therapy in children? Curr Opin HIV AIDS 2007 14 426 430 10.1097/COH.0b013e3282ced12c 19372922

[B8] Sohn AH Ananworanich J Highly active antiretroviral therapy for children with treatment failure HIV Ther 2009 14 485 499 10.2217/hiv.09.28

[B9] WHO Antiretroviral therapy for HIV infection in infants and children: Towards universal access: Recommendations for a public health approach-2010 revision. Geneva 2010 23741772

[B10] WHO Antiretroviral therapy of HIV infection in infants and children in resource-limited settings: Towards universal access, Recommendations for a public-health approach. Geneva 2006 23741772

[B11] Janssens B Raleigh B Soeung S Akao K Te V Gupta J Vun MC Ford N Nouhin J Nerrienet E Effectiveness of highly active antiretroviral therapy in HIV-positive children: evaluation at 12 months in a routine program in Cambodia Pediatrics 2007 14 e1134 1140 10.1542/peds.2006-3503 17954553

[B12] Zhang F Haberer JE Zhao Y Dou Z Zhao H He Y Cao GH Chinese pediatric highly active antiretroviral therapy observational cohort: a 1-year analysis of clinical, immunologic, and virologic outcomes J Acquir Immune Defic Syndr 2007 14 594 598 10.1097/QAI.0b013e318158c08e 18043313

[B13] Davies MA Wood R Van Cutsem G Giddy J Eley B Rabie H Moultrie H Technau K Boulle A (IeDEA-SA) Virologic failure and second-line antiretroviral therapy (ART) in children in South Africa: the international epidemiologic databases to evaluate AIDS (IeDEA) Southern Africa collaboration J Acquir Immune Defic Syndr 2010 in press 10.1097/QAI.0b013e3182060610 PMC3104241 21107266

[B14] Kamya MR Mayanja-Kizza H Kambugu A Bakeera-Kitaka S Semitala F Mwebaze-Songa P Castelnuovo B Schaefer P Spacek LA Gasasira AF Katabira E Colebunders R Quinn TC Ronald A Thomas DL Kekitiinwa A Academic Alliance for AIDS Care and Prevention in Africa Predictors of long-term viral failure among Ugandan children and adults treated with antiretroviral therapy J Acquir Immune Defic Syndr 2007 14 187 193 10.1097/QAI.0b013e31814278c0 17693883

[B15] WHO Rapid advice: Antiretroviral therapy for HIV infection in adults and adolescents. Geneva 2009

[B16] Aurpibul L Puthanakit T Lee B Mangklabruks A Sirisanthana T Sirisanthana V Lipodystrophy and metabolic changes in HIV-infected children on non-nucleoside reverse transcriptase inhibitor-based antiretroviral therapy Antivir Ther 2007 14 1247 1254 18240864

[B17] WHO Report of the WHO Technical Reference Group, Paediatric HIV/ART Care Guideline Group Meeting. Geneva 2008

