# A biregional survey and review of first-line treatment failure and second-line pediatric antiretroviral access and use in Asia and southern Africa

**DOI:** 10.1186/1758-2652-14-17

**Published:** 2011-04-08

**Affiliations:** 1 TREAT Asia/amfAR - The Foundation for AIDS Research, Bangkok, Thailand; 2 Institute of Social and Preventive Medicine, University of Bern, Bern, Switzerland and the School of Public Health and Family Medicine, University of Cape Town, Cape Town, South Africa

## Correction

The article published in [1], contains incorrect data in Table 1 regarding the nationally recommended paediatric second-line ART regimen after NNRTI failure in India. This has been corrected. The editors and authors regret the inconvenience caused to readers by the error in the original paper.

**Table 1:** First- and second-line antiretroviral therapy regimens in use in TREAT Asia and IeDEA Southern Africa

Site	Country	Nationally recommended paediatric first-line ART regimen*	Nationally recommended paediatric second-line ART regimen after NNRTI	Most commonly used second-line regimen in current site cohort
**TREAT Asia**				

National Center for HIV, AIDS, Dermatology, and Sexually Transmitted Infections	Cambodia	d4T or AZT+3TC+NVP or EFV If NNRTI exposure: d4T+3TC+LPV/r	ABC+ ddI+LPV/r	ABC+3TC+LPV/r

Beijing Ditan Hospital	China	d4T or AZT+3TC+NVP or EFV If NNRTI exposure: AZT+3TC+LPV/r	ABC+3TC+AZT+LPV/r	ABC+3TC+AZT+LPV/r

YRG Centre for AIDS Research and Education	India	d4T or AZT+3TC+NVP or EFV	ABC+ddI+3TC+LPV/r	TDF or ddI+3TC or FTC+LPV/r

Cipto Mangunkusumo Hospital	Indonesia	d4T or AZT+3TC+NVP or EFV	ddI+3TC+LPV/r	ddI+3TC+LPV/r

Hospital Kuala Lumpur	Malaysia	<3yr: AZT+3TC or ddI+NVP ≥3yr: AZT+3TC or ddI+EFV	2 new NRTI+LPV/r	d4T+3TC+LPV/r

Hospital Likas	Malaysia	<3yr: AZT+3TC or ddI+NVP ≥3yr: AZT+3TC or ddI+EFV	2 new NRTI+LPV/r	d4T+ddI+LPV/r

Hospital Penang	Malaysia	<3yr: AZT+3TC or ddI+NVP ≥3yr: AZT+3TC or ddI+EFV	2 new NRTI+LPV/r	--

Hospital Raja Perempuan Zainab	Malaysia	<3yr: AZT+3TC or ddI+NVP ≥3yr: AZT+3TC or ddI+EFV	2 new NRTI+LPV/r	d4T+ddI+LPV/r

Chiang Mai University Medical Centre	Thailand	d4T or AZT+3TC+NVP or EFV	ddI+ABC or 3TC+PI/r	AZT+3TC+LPV/r

Chiang Rai Regional Hospital	Thailand	d4T or AZT+3TC+NVP or EFV	ddI+ABC or 3TC+PI/r	AZT+3TC+LPV/r

HIV-NAT	Thailand	d4T or AZT+3TC+NVP or EFV	ddI+ABC or 3TC+PI/r	AZT+3TC+LPV/r

Khon Kaen University Medical Centre	Thailand	d4T or AZT+3TC+NVP or EFV	ddI+ABC or 3TC+PI/r	AZT+ddI+LPV/r

Siriraj Hospital	Thailand	d4T or AZT+3TC+NVP or EFV	ddI+ABC or 3TC+PI/r	AZT+ddI+LPV/r

Children's Hospital 1	Vietnam	d4T+3TC+NVP	ABC+ddI+LPV/r	--

Children's Hospital 2	Vietnam	d4T+3TC+NVP	ABC+ddI+LPV/r	ABC+ddI+LPV/r

National Hospital of Paediatrics	Vietnam	d4T+3TC+NVP	ABC+ddI+LPV/r	ABC+ddI+LPV/r

**IeDEA Southern Africa**				

Lighthouse Clinic	Malawi	d4T+3TC+NVP	ABC+ddI+LPV/r	AZT+3TC+LPV/r

Paediatric Day Hospital, Maputo	Mozambique	d4T or AZT+3TC+NVP or EFV If NNRTI exposure: d4T or AZT+3TC+LPV/r	ABC+ddI+LPV/r	None

Rahima Moosa Mother and Child Hospital	South Africa	<3yr/10kg: d4T+3TC+LPV/r >3yr/10kg: d4T+3TC+EFV	AZT+ddI+LPV/r	ABC+3TC+LPV/r

Gugulethu Community Health Centre	South Africa	<3yr/10kg: d4T+3TC+LPV/r >3yr/10kg: d4T+3TC+EFV	AZT+ddI+LPV/r	AZT+ddI+EFV or LPV/r ABC+3TC+LPV/r

Harriet Shezi Clinic	South Africa	<3yr/10kg: d4T+3TC+LPV/r >3yr/10kg: d4T+3TC+EFV	AZT+ddI+LPV/r	AZT+ddI+LPV/r

Khayelitsha Community Health Centre	South Africa	<3yr/10kg: d4T+3TC+LPV/r >3yr/10kg: d4T+3TC+EFV	AZT+ddI+LPV/r	AZT+ddI+LPV/r

McCord Hospital	South Africa	<3yr/10kg: d4T+3TC+LPV/r >3yr/10kg: d4T+3TC+EFV	AZT+ddI+LPV/r	AZT+ddI+LPV/r

Red Cross Children's Hospital	South Africa	<3yr/10kg: d4T+3TC+LPV/r >3yr/10kg: d4T+3TC+EFV	AZT+ddI+LPV/r	AZT+ddI+EFV

Tygerberg Hospital	South Africa	<3yr/10kg: d4T+3TC+LPV/r >3yr/10kg: d4T+3TC+EFV	AZT+ddI+LPV/r	AZT+ddI+LPV/r

Newlands Clinic	Zimbabwe	AZT+3TC+NVP	ddI+3TC+LPV/r	d4T+3TC+NVP

*Content reflects current recommendations at the time of the survey. WHO first-line regimen recommendations at the time of the survey included two NRTIs with one NNRTI or two NRTIs with one PI/r if the infant had previous NNRTI exposure [10,17]; second-line regimen recommendations after NNRTI failure included two NRTIs with one PI/r or unboosted nelfinavir in limited circumstances.

d4T - stavudine; AZT - zidovudine; 3TC - lamivudine; NVP - nevirapine; EFV - efavirenz; ABC - abacavir; ddI - didanosine; LPV/r - ritonavir-boosted lopinavir; TDF - tenofovir; FTC - emtricitabine; NRTI - nucleoside reverse transcriptase inhibitor; NNRTI - non-nucleoside reverse transcriptase inhibitor.

## References

[B1] TREAT Asia Pediatric HIV Observational Database (TApHOD), and The International Epidemiologic Databases to Evaluate AIDS (IeDEA) Southern Africa Paediatric Group A biregional survey and review of first-line treatment failure and second-line paediatric antiretroviral access and use in Asia and southern Africa J Int AIDS Soc 2011 14 1 7 21306608 10.1186/1758-2652-14-7 PMC3058075

